# Bacterial Type II Secretion System and Its Mitochondrial Counterpart

**DOI:** 10.1128/mbio.03145-22

**Published:** 2023-03-27

**Authors:** Anna Shaliutina-Loginova, Olivera Francetic, Pavel Doležal

**Affiliations:** a Department of Parasitology, Faculty of Science, BIOCEV, Charles University, Vestec, Czech Republic; b University of South Bohemia in České Budějovice, Faculty of Fisheries and Protection of Waters, South Bohemian Research Center of Aquaculture and Biodiversity of Hydrocenoses, Research Institute of Fish Culture and Hydrobiology, Vodňany, Czech Republic; c Institut Pasteur, Université Paris Cité, Biochemistry of Macromolecular Interactions Unit, Department of Structural Biology and Chemistry, CNRS UMR 3528, Paris, France; University of Michigan Medical School; Ohio State University

**Keywords:** type II secretion system, protein secretion, mitochondrial evolution, T2SS, evolution, mitochondria, protein transport

## Abstract

Over the billions of years that bacteria have been around, they have evolved several sophisticated protein secretion nanomachines to deliver toxins, hydrolytic enzymes, and effector proteins into their environments. Of these, the type II secretion system (T2SS) is used by Gram-negative bacteria to export a wide range of folded proteins from the periplasm across the outer membrane. Recent findings have demonstrated that components of the T2SS are localized in mitochondria of some eukaryotic lineages, and their behavior is consistent with the presence of a mitochondrial T2SS-derived system (miT2SS). This review focuses on recent advances in the field and discusses open questions concerning the function and evolution of miT2SSs.

## INTRODUCTION

Bacteria can use secretion systems to transport individual proteins and protein complexes, as well as DNA-protein complexes, into adjacent cells or the extracellular environment. Protein secretion systems are critical for bacterial survival and adaptation to complex environmental conditions through their diversified secreted effectors (exoproteins), which are required for nutrient uptake, toxin delivery, cell-to-cell communication, and interspecies competition. At least 10 types of secretion systems have been identified in Gram-negative and in some Gram-positive bacteria containing an outer membrane layer (1). They differ in origin, architecture, energizing mechanism, and the type of protein cargo they carry across one, two, or even three membranes (the outermost membrane belonging to a target cell). Some secretion systems are found in almost all bacteria (e.g., type I secretion systems [T1SS] or T6SS) and secrete a wide variety of substrates, while others are limited to only a few bacterial species or are dedicated to secreting only one or a few proteins. Due to their complexity, function in specific environments, diversity of their substrates, and overall dynamics, there are still many unknowns regarding the mechanisms and precise biological roles of these sophisticated nanomachines.

Type II protein secretion systems are one of the best-characterized bacterial protein translocation machines that promote the specific transport of folded proteins across a dedicated channel in the outer membrane. The T2SS is particularly concentrated in the alpha-, beta-, gamma-, and deltaproteobacteria, as well as the *Bacteroidetes* and *Deferribacteres* (2, 3), and it represents a key virulence factor in many human pathogens, including Acinetobacter baumannii (4), Vibrio cholerae (5), and Legionella pneumophila (6). In many bacteria, the T2SS secretes a broad repertoire of substrates that are released into the milieu or displayed on the cell surface, contributing to bacterial adaptation to a wide range of habitats. All this affords the T2SS a fundamental importance as a key mechanism by which bacteria induce local and global modifications of their external environment (7).

Eukaryotes have been considered devoid of the T2SS and other systems that secrete proteins to the surface of diderm bacteria, despite the bacterial roots of mitochondria and plastids, which are of endosymbiotic origin (8, 9). Indeed, the vast majority of mitochondria of extant eukaryotes carry only rudiments of the original bacterial protein transport machines that have very limited functions and do not mediate secretion of proteins (10). While this situation is a product of billions of years of independent eukaryotic evolution, it is very likely that the bacterial ancestor of mitochondria, derived from the alphaproteobacterial endosymbiont, was secreting proteins via the T2SS to the host cell, as in the case of current intracellular bacteria. The nature of metabolic relationships between the mitochondrial ancestor and the archaeal host has been debated for years, and different possible syntrophic interactions have been proposed (11). The secretion of proteins by the endosymbiont may have played a crucial role in establishing this interaction that gave rise not only to the mitochondrion and but very likely to the eukaryotic cell itself. Interestingly, genes encoding components of the T2SS pathway have been recently discovered in several eukaryotic lineages, and mitochondrial localization of some of these proteins has been demonstrated (12, 13). Phylogenetic distribution of these genes suggests that the T2SS was used by the last eukaryotic common ancestor (LECA) and that therefore its characterization may speak about the early evolutionary history of the eukaryotic cells.

This review presents the general architecture and function of the T2SS of current free-living and pathogenic bacteria. It discusses the available information on the newly discovered mitochondrial counterpart of the T2SS and highlights the similarities and differences between the two pathways. Finally, it proposes a hypothesis on the role of the T2SS during the evolution of eukaryotes.

## GENERAL OVERVIEW OF T2SS

### Origins of T2SS.

The T2SS evolved from an ancestral nanomachine that also gave rise to the systems that assemble type IV pili (T4P) in bacteria, as well as the flagella, pili, and other surface structures in archaea (3). According to the phylogeny of common components, it is very likely that the core structure of T2SS was already present in the last universal common ancestor (LUCA). Both bacterial and archaeal nanomachines of this superfamily follow an analogous blueprint yet are accompanied by lineage-specific adaptations (3). They are involved in multiple functions, including cell adherence, aggregation, macromolecular trafficking, formation of biofilms, and motility (14, 15). They all build proteinaceous filaments anchored in the inner membrane that can extend or retract by the addition or removal of matured subunits. In most T4P, these subunits are the only substrates of the assembly machinery which allows the pili to pass through the outer membrane channel and reach beyond the cell surface (16). The T2SS also encompasses a pilus-like structure, referred to as the pseudopilus or endopilus, since it is limited to the periplasmic space (17). Similar to T4P, the machinery is responsible for the endopilus extension and possibly passive retraction, but its actual substrates are specific protein cargo that the endopilus drives through the outer membrane channel. Different models have been proposed for the endopilus mode of action; however, none of them has been fully validated (17).

### Architecture of the T2SS.

**FIG 1:**
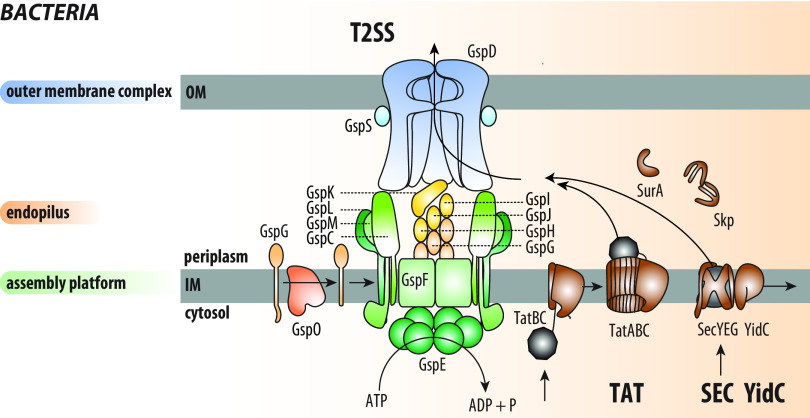
T2SS machinery in Gram-negative bacteria. Secretion of proteins via the T2SS from bacterial cytoplasm starts with translocation across the inner (cytoplasmic) membrane. This step is mediated either via Sec or Tat translocases that transport unfolded or folded proteins, respectively. The substrates of the T2SS (shown in gray) are loaded on the tip of the endopilus (orange), assembly of which occurs at the assembly platform (AP; green) by the maturation of GspG subunits by the prepilin peptidase GspO. The interaction of the substrate with the periplasmic domains of GspD (blue) induces the pore opening and the release of the substrate. The secretion is energized by GspE ATPase. Most proteins reach the periplasm in an unfolded state via the SecYEG translocase, which cooperates with YidC for the insertion of inner membrane proteins. TatABC constitutes transient translocation pores for folded substrates. Folding and unfolding in the periplasm is mediated by SurA and Skp chaperones. IM, inner (cytoplasmic) membrane; OM, outer membrane.

The T2SS multiprotein complex, also known as the terminal branch of the general secretion pathway (GSP) is encoded by genes often arranged in a single operon, named *gspC* to *gspO* (7). The T2SS can be divided into three subcomplexes (Fig. 1), coordinate action of which enables the transport of the cargo: (i) an outer membrane complex (OMC), which includes the secretin channel formed by GspD and the pilotin GspS and is connected via GspC to (ii) an inner membrane complex called the assembly platform (AP), including GspF, GspL, and GspM and the cytoplasmic ATPase GspE, and (iii) the endopilus, composed of the major pilin GspG and the minor pilins GspH, GspI, GspJ, and GspK. GspO, the prepilin peptidase embedded in the inner membrane, processes the five pilins into a mature form competent for membrane extraction and endopilus assembly (18, 19).

In some systems, the secretin biogenesis is supported by additional factors in the inner membrane, including GspA and GspB. The inner membrane protein GspB binds to peptidoglycan and interacts with the N-terminal domain of GspD, thereby assisting its outer membrane insertion (20). GspB acts in concert with GspS or with the ATPase GspA, which is present in a subset of species (21). GspN is found only in some T2SS and forms part of the AP within the inner membrane (22).

*In vitro* structural studies of purified components and subcomplexes have significantly improved our understanding of the T2SS (23). X-ray crystallography and nuclear magnetic resonance have provided a wealth of information on soluble components or domains, and recent advances in electron microscopy (EM) have enabled studies of larger subcomplexes. Integrative approaches have been employed to understand how the different components of the T2SS are positioned and how they interact with each other within an intact cell envelope. The recent work by Ghosal et al. (24) provided the first comprehensive view of a T2SS assembled *in situ*, and those researchers traced a topology map to position most components. High-resolution cryo-EM studies revealed the detailed structure of the OMC, including the pentadecameric assembly of GspD and GspS (22, 25, 26). The secretin domain of GspD builds the membrane β-barrel and the periplasmic gate, while the N-terminal N0 to N3 domains protrude into the periplasm, where they interact with GspB (20) and GspC, which may constitute a pore plug (22), and possibly with the exoprotein substrates. The C-terminal S-domain of GspD interacts with the pilotin, a small lipoprotein that mediates the targeting of GspD to the outer membrane.

The AP is built around an oligomer of the polytopic IM protein GspF (27), while GspL and GspM proteins of the subcomplex carry single transmembrane segments and expose their ferredoxin-like domains to the periplasm (28). GspC links the AP to the N-terminal domain of GspD (23, 29). The cytoplasmic hexamer of GspE ATPase acts as the motor for the secretion system (30). It comprises N1, N2, and the C-terminal domain, which contains the nucleotide-binding and hydrolysis Walker A and B motifs (31). The N1 domain anchors GspE to the AP though its direct interaction with the cytoplasmic domain of GspL (27). The endopilus is a helical assembly of the major pilin GspG (32, 33), with the tip composed of minor pilins GspHIJK that initiate the assembly (34, 35).

These structural and functional data have offered the possibility to propose a functional model of protein secretion via T2SS. The endopilus assembly might be constitutive or triggered by binding of the cargo to AP components or to the minor pilins (36). The addition of new GspG subunits would extend the endopilus and allow the cargo to enter secretin cavity by inducing conformational changes of the GspD N domains. The opening of the periplasmic gate would enable the release of the cargo into the outer chamber of the secretin channel and its diffusion to the cell surface.

### T2SS substrates.

The T2SS substrates are initially transported across the inner membrane by the Sec or Tat systems (Fig. 1) (37). The two pathways converge in the periplasm to form a pool of folded intermediates. The Tat-dependent substrates enter this pool in a prefolded state and often carry cofactors, while the Sec-dependent proteins fold in the oxidative periplasmic space and are stabilized by calcium ions and covalent disulfide bonds. Here comes the main advantage of the T2SS: it secretes bulky, prefolded and preassembled cargo that can readily exert functions in the harsh external environments. All these substrates differ among taxa and share no common sequence or structural features (23), raising a question on their recognition mechanisms, which are still unresolved.

The current assumption is that there are one or more conformational signals of the folded cargo composed of several motifs spread along the primary amino acid sequence of the protein. In this manner, the secretion signal may be composed of residues from different locations in the linear polypeptide chain, which may or may not be brought together into a conformational patch upon protein folding (36, 38). Resent structure-based studies have favored the presence of multiple signals present in flexible or even unstructured regions of exoproteins (39, 40).

The presence of conformational signals is especially intriguing, given their specific and possibly sequential interactions with the different T2SS components, including secretin (41), the endopilus tip (36), or the AP subunits (42). The substrates are released to the milieu or remain associated with the cell surface (43). The latter include lipidated proteins (39, 44) or proteins that associate with the lipopolysaccharide (45). Alternatively, the anchor can be formed by an unprocessed Tat signal peptide (46).

The proteins released by the T2SS include multiple, structurally unrelated, species-specific factors, such as toxins, hydrolases, lipid-modifying enzymes, and phosphatases (43). Their number and repertoire vary vastly between environmental and commensal bacteria and between extracellular or intracellular pathogens. It is therefore highly likely that the coevolution of the T2SS machines together with their cognate substrates has led to a gradual adaptation to achieve the optimal fit.

## ORIGIN OF MITOCHONDRIA AND THE EXPORT OF PROTEINS

One such dramatic evolutionary event occurred when a bacterial endosymbiont belonging to alphaproteobacteria or to their relatives (47, 48) underwent a transformation into the mitochondrion. It is very likely that the T2SS and other protein export pathways of the bacterial endosymbiont played a crucial role in establishing its relationship with the host cell that continues to this day (49). Until recently, however, there was no concrete evidence for this scenario.

In general, several examples document that protein transport machines of bacterial origin can function in mitochondria. Mitochondria of extant eukaryotes still employ orthologues of bacterial protein translocases OxaI and Sam50, which mediate the insertion of the α-helical or β-barrel membrane proteins into the inner or outer mitochondrial membrane, respectively (10, 50). Some have preserved Tat and Sec translocases in the inner membrane, the functions of which, however, remain unknown or might be dedicated to a single known protein substrate (51–53).

To date, no protein is known to be actively exported from the mitochondria. Several proteins leave mitochondria during apoptosis, but as passive cargos released from the intermembrane space upon the outer membrane permeabilization by Bcl-2 family members (49). Interestingly, recent years have also brought reports on the formation of vesicles of the mitochondrial outer membrane that may fuse with peroxisomes (54) or even serve as a basis for peroxisome biogenesis (55). This process is reminiscent of the outer membrane vesicle secretion by bacteria (56).

The absence of bacterial secretion systems in mitochondria is in direct contrast to the situation of current alphaproteobacteria, most of which carry the T2SS (2, 3). The abundance of the T2SS among alphaproteobacterial species has indicated that the secretion pathway was present in the arsenal of the bacterial ancestor of mitochondria (3). This would suggest that either the T2SS was not compatible with mitochondrial physiology or simply not needed anymore and was lost during the endosymbiont transition to mitochondria. Alternatively, it remained present in a limited set of eukaryotes evolutionarily distant from the main experimental models, such as yeast and mammalian cells. The recent discovery of a mitochondrial T2SS (miT2SS) pathway in diverse groups of unicellular eukaryotes argues for the latter and provides an additional piece in the puzzle of the prokaryote-to-mitochondrion transition.

### Mitochondrial T2SS: adaptation, not just a remnant.

**FIG 2:**
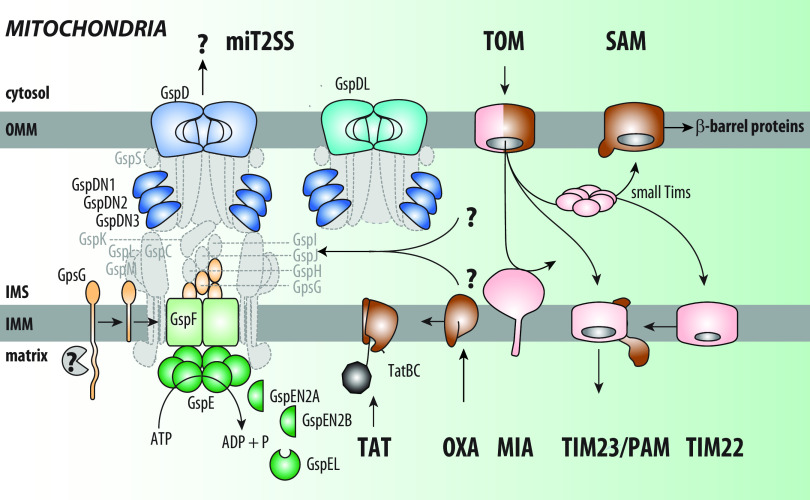
Mitochondrial counterpart of the T2SS. Eukaryotic homologues of T2SS components could constitute a mitochondrial secretion pathway (miT2SS). They include core components of all subcomplexes of bacterial pathways, depicted using the same color code as in Fig. 1, including two GspD proteins (GspD and GspDL) of the outer membrane pore, endopilus subunit GspG, and AP proteins GspF and GspE. While eukaryotic GspD contains only the membrane-embedded secretin domain of the bacterial homologue, three eukaryote-specific proteins (GspDN1 to -3) carry homology to soluble N domains of bacterial GspD. Three additional proteins related to bacterial GspE (GspEN2A, GspEN2B, and GspEL) were also identified in eukaryotes. The two question marks indicate that substrates transported by miT2SS could arrive to the intermembrane space (IMS) either from cytosol (via TOM complex) or the mitochondrial matrix. Mitochondrial GspG was suggested to be imported from the cytosol (via TIM23 complex) with a long presequence that is cleaved upon import by the mitochondrial processing peptidase or some other protease (labeled by question mark). Mitochondrial protein transport pathways present in the heterolobosean Naegleria gruberi are shown for the complete picture. Translocases with clear bacterial ancestry are shown in brown, the rest (eukaryote specific) are depicted in pink. Note that the bacterial origin of the core TOM complex subunit (Tom40) is suggested but remains without a clear bacterial homologue. IMM, inner mitochondrial membrane; OMM, outer mitochondrial membrane; IMS, intermembrane space. The components missing in miT2SS relative to the bacterial T2SS are depicted in gray.

The inspection of distant eukaryotic species from the classes *Heterolobosea*, *Jakobida*, *Malawimonadida*, and *Hemimastigophora*, which are all heterotrophic protists, revealed the unexpected presence of sequences orthologous to the genes encoding core T2SS subunits (12, 13). Initial identification of *gspG* homologues in their genomes led to the thorough analysis directed at the rest of T2SS machinery. Interestingly, these included all genes encoding subunits central to all three functional subcomplexes: the outer and the inner membrane complexes and the endopilus (Fig. 2).

The identified orthologues are highly divergent from all bacterial sequences, which complicated the identification of their closest relatives and the original bacterial ancestor. On the other hand, all eukaryotic sequences are mutually very similar, despite the evolutionary distance of the eukaryotic owners, and share the same domain features that are different from the bacterial T2SS. This strongly suggests that these eukaryotes carry the same ancestral secretion system that was once operating in the LECA. Importantly, several components of miT2SS have been localized to mitochondria, as revealed by immunofluorescence microscopy and proteomics, where they likely assemble into a secretion supercomplex (12).

While the biochemical *in situ* evidence is missing, according to the sequence analyses, the experimental heterologous expression in Escherichia coli, and the *in vitro* import assay into isolated yeast mitochondria, the outer membrane complex is present in the outer mitochondrial membrane (12). There have been five genes identified in eukaryotes that are partially homologous to bacterial *gspD* (Fig. 2). Two genes encode the C-terminal part of the C domain (GspD and GspDL) and three encode short sequences corresponding to the N domains of bacterial GspD (GspDN1 to -3). Thus, the mitochondrial outer membrane channel may be a multimer formed by five independent protein types or their subsets, whereas the bacterial pore is an oligomer of a single polypeptide. A single gene encoding a GspF homologue has been identified in eukaryotes, which would make it the only component of the mitochondrial inner membrane assembly platform (AP). Like its bacterial counterpart, GspF may exist as an oligomer as suggested by its self-interaction in two-hybrid assays (12). Orthologues of genes encoding other inner membrane AP subunits GspL, GspM, and GspC are missing in the eukaryotic genomes or may have significantly diverged, precluding their identification. Alternatively, other miT2SS components or domains might have taken over their roles. This might link to the multiplication of gene copies for other subunits. Eukaryotes carry one clear orthologue of *gspE* encoding the ATPase, plus three additional sequences that seem to be evolutionarily derived. One would encode just the nucleotide binding domain, yet with the abrogated ATPase motif (GspEL), and two relate to the N-terminal domains of GspE (GspEN2A and GspEN2B). Such a combination of components indicates that the latter proteins regulate the activity of the GspE orthologue. The GspE orthologue lacking the ATPase motif is reminiscent of PilM protein in T4P systems, itself an orthologue of the cytoplasmic domain of GspL (57), supporting this possibility.

The functional principle of the secretion driven by T2SS is the assembly of major pilin subunits GspG. Genes encoding its orthologues are present in eukaryotic genomes, and GspG localization in mitochondria was confirmed by microscopy and proteomics (12). The mitochondrial GspG carries large nonhomologous N-terminal sequence that is likely processed *in vivo*, and the resulting mature protein then corresponds to its bacterial counterpart. The genes encoding regulatory but essential minor pilins GspH, GspI, GspJ, and GspK and the prepilin peptidase GspO are absent in eukaryotes. This might reflect the differences in protein biogenesis and localization pathways that impose novel constraints to miT2SS assembly. The large N-terminal extension of the eukaryotic pre-GspG probably carries a mitochondrial import and inner membrane targeting signals, and possibly other functions. The very presence of the N-terminal targeting sequences in GspG and GspF argues in favor of a functional miT2SS: in the process of gene migration to the host cell nucleus, the miT2SS components have acquired signals allowing them to regain their original mitochondrial localization required for function. In the case of pre-GspG, this path is particularly lengthy, from the cytoplasmic ribosomes through import into mitochondria across the OM and insertion into the IM, followed by membrane extraction and polymerization in the intermembrane space (12). The proteolytic activity of GspO, essential in bacteria and archaea, may be compensated by another protease capable of a specific cleavage following the His residue near the membrane-cytoplasmic interface. Such cleavage would reveal the positively charged N terminus of the mature miGspG, which binds to negatively charged phospholipids and prevents its membrane extraction. The presence of the conserved residue E5, which neutralizes this positive charge (19), suggests that this critical step of bacterial endopilus polymerization is conserved in mitochondrial T2SS. The absence of minor pilins that presumably form a tip complex is more difficult to explain, as this complex initiates endopilus polymerization in bacterial T2SS and possibly serves as a substrate binding epitope. Other mitochondrial proteins distinct from bacterial counterparts might have taken over these functions.

Although many essential subunits of bacterial T2SS are missing in the miT2SS, there are two aspects that support the functionality of the latter despite the lack of direct functional characterization (12). First, diverse groups of eukaryotes (*Heterolobosea*, *Jakobida*, *Malawimonadida*, and *Hemimastigophora*) share exactly the same set of components and not just bits and pieces of domains abandoned in the genome. Second, these conserved genes encode the core components of all functional units (subassemblies) found in bacterial T2SS that may build the secretion apparatus in its minimalist design. On the other hand, the miT2SS may accommodate eukaryote-specific components that were added during the functional adaptation in the early mitochondrion.

### Extension of miT2SS.

Phylogenomic profiling is a powerful approach that weighs the joint presence or absence of particular traits across a large number of species (58). Such analyses can identify novel biological functions or pathways that require concerted actions of multiple proteins which are collectively absent in species lacking the pathway. Using this approach, eukaryotic genomes encoding miT2SS were searched for other exclusive components that are missing in other organisms. These analyses generated a set of 16 genes encoding Gsp cooccurring proteins (Gcps). Due to the nature of the phylogenetic profiling, most of the identified proteins lacked homology to known protein families, yet they carried sequence motifs such as WD40 repeats or conserved cysteine-histidine residues, which suggest metal-coordination properties (12).

Bioinformatic and proteomic analyses suggested that Gcps are either mitochondrial or peroxisomal proteins, suggesting a link between miT2SS and peroxisome function. However, functional analyses are needed to determine the localization of Gcps and their actual connection to miT2SS.

## CONCLUSIONS

### Understanding the function of miT2SS.

Given the great variability among the T2SS substrates and the lack of unified secretion signals, it is currently impossible to predict the mitochondrial substrates of miT2SS and, hence, the function of mitochondrial T2SS preserved in several unicellular lineages of eukaryotes. Importantly, this pathway may be nonessential and has been lost in most existing eukaryotes. Given our thorough knowledge of mitochondrial metabolism in mammals and fungi, it is more likely that whatever substrate miT2SS secreted was no longer required in these organisms or its production was relocated to a different cellular compartment.

Currently, the only way to understand the function of miT2SS is through *in situ* experimental studies directly in these eukaryotes (*Heterolobosea*, *Jakobida*, *Malawimonadida*, and *Hemimastigophora*). Of them, only heteroloboesan amoebae are available for axenic cultures that allow biochemical characterization of the pathway (59). Naegleria gruberi is the most studied heterelobosean with available genome data (60) that is used as a valuable cell biology model (61, 62). In addition, it serves as a nonpathogenic model to understand the biology of distantly related Naegleria fowlerii, a causative agent of fatal primary amoebic meningoencephalitis (63). Despite considerable efforts of many labs, the experimental toolbox for *N. gruberi* and other heteroloboseans remains very limited, as reliable reverse genetics approaches are lacking, which would allow researchers to dissect the molecular details of the secretion system. Therefore, experimental studies must rely on biochemical methods, such as spatial proteomics, including hyperplexed localization of organelle proteins by isotope tagging (i.e., DC-LOPIT) (64) and sensitive *in organello* metabolic experiments that could capture proteins or other biomolecules exported from the mitochondria. Moreover, the complexity of the machinery and its altered biogenesis pathways do not really allow the transfer of miT2SS into heterologous bacterial systems. In parallel, improved resolution of cryo-electron tomography might provide structural insights into these systems *in situ* (65).

Based on the set of genes identified by phylogenetic profiling (encoding Gcps) that coevolved with miT2SS, a hypothesis was formulated proposing that mitochondria may produce a cofactor-containing protein that is matured and exported to peroxisomes. Mitochondria play an essential role in the synthesis of copper and iron-containing prosthetic groups. Products of the iron-sulfur cluster biosynthesis pathway are also exported from mitochondria to become incorporated into specific proteins in the cytosol and other eukaryotic compartments (66). Mitochondria harbor the final steps of heme synthesis, and its export from mitochondria remains unclear (67). Similarly, the mitochondrial intermembrane space machinery mediates the insertion of copper atoms into the cytochrome oxidase complex (68).

Hence, it is plausible that an iron- or copper-containing holoprotein is assembled or matured via a mitochondria-based pathway and exported via miT2SS to peroxisomes. But where is this unknown cargo coming from? It is possible that such a protein is encoded in the mitochondrial genome and translated on the mitoribosome. While mitochondrial genomes of *N. gruberi* and other eukaryotes with miT2SS encode in fact more proteins than in metazoans and other eukaryotes (69), there is no sequence in the mitochondrial genomes that would be a good candidate for the proposed maturation and export scenario. Therefore, in an alternative model, the protein is first imported into mitochondria, where it is functionalized via a prosthetic group and exported from the organelle (12). As exciting as this hypothesis seems, it will require considerable experimental work to validate it and to understand why some eukaryotes retained this ancestral complex trait.
